# O.2.2-1 Shining a light on success: the impact of the ‘Ireland Lights Up’ walking initiative on health and wellbeing

**DOI:** 10.1093/eurpub/ckad133.116

**Published:** 2023-09-11

**Authors:** Barry Lambe, Aisling McGrath, Niamh Murphy, Nicola Briggs

**Affiliations:** South East Technological University, Ireland; barry.lambe@setu.ie; South East Technological University, Ireland; barry.lambe@setu.ie; South East Technological University, Ireland; barry.lambe@setu.ie; South East Technological University, Ireland; barry.lambe@setu.ie

## Abstract

**Purpose:**

‘Ireland Lights UP’ (ILU) is an annual walking initiative implemented by the Gaelic Athletic Association (GAA) and several other partners in Ireland. The initiative sees more than 1,000 GAA clubs turning on their floodlights for the local community to walk around their grounds over a 7-week period beginning in January. An estimated 35,000 participants take part annually. The initiative has the potential to create sustainable improvements to multiple aspects of physical, social and mental wellbeing. However, there has been limited evaluation of ILU.

**Methods:**

This was a mixed methods study consisting of surveys with adult ILU participants in 12 GAA clubs (n = 181) and semi-structured qualitative interviews with ILU co-ordinators at both club (n = 12) and national level (n = 1). Cross-sectional surveys (six Likert scale questions assessing participation and impact on wellbeing) were administrated via an online link to an MS Form or via a structured interview. The qualitative interviews were conducted using zoom, structured using a hybrid of the RE-AIM and PRISM frameworks and analysed using thematic content analysis.

Results: The average age of survey respondents was 52.3 years and the majority (73%) were female. Almost 60% of respondents reported that they mostly walked with friends and 77% attended more than half of the organised walks. A high proportion of respondents strongly agreed that they exercised more because of ILU (56.4%), spoke to people they would not normally speak to (45.5%) and intended to maintain their increased walking (54.3%). Almost 66% stated that ILU improved their general health and wellbeing. The key themes elicited from the interviews were narratives around the lasting benefits of ILU, the restoration of connections between people, ring-fencing of quality time with friends and family and increasing social capital and connectedness.

**Conclusions:**

Ireland Lights Up is a rare example of a PA programme already operating at scale. The standout benefit of ILU was the creation of social connections at a time when daylight hours are short and PA levels are lower. This study was formative in nature and will help to shape the research design of a more comprehensive evaluation of the implementation of ILU.

**Support/funding source:**

non applicable.

